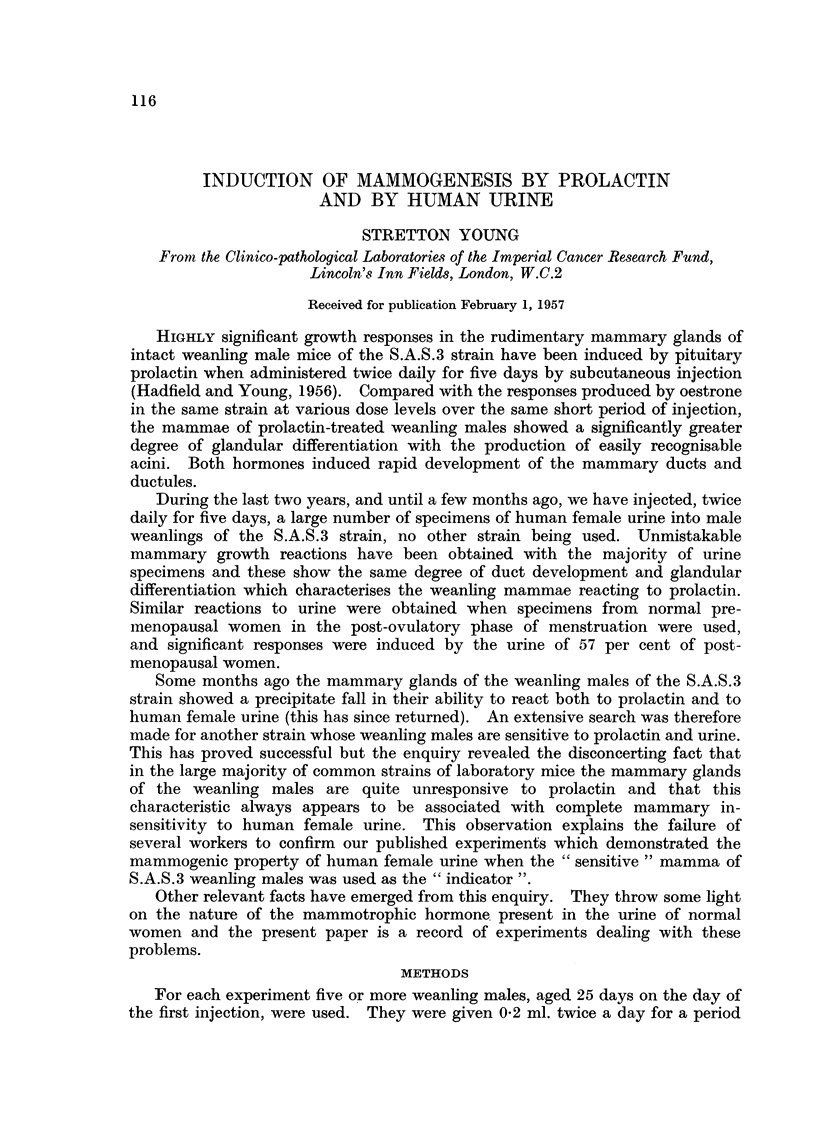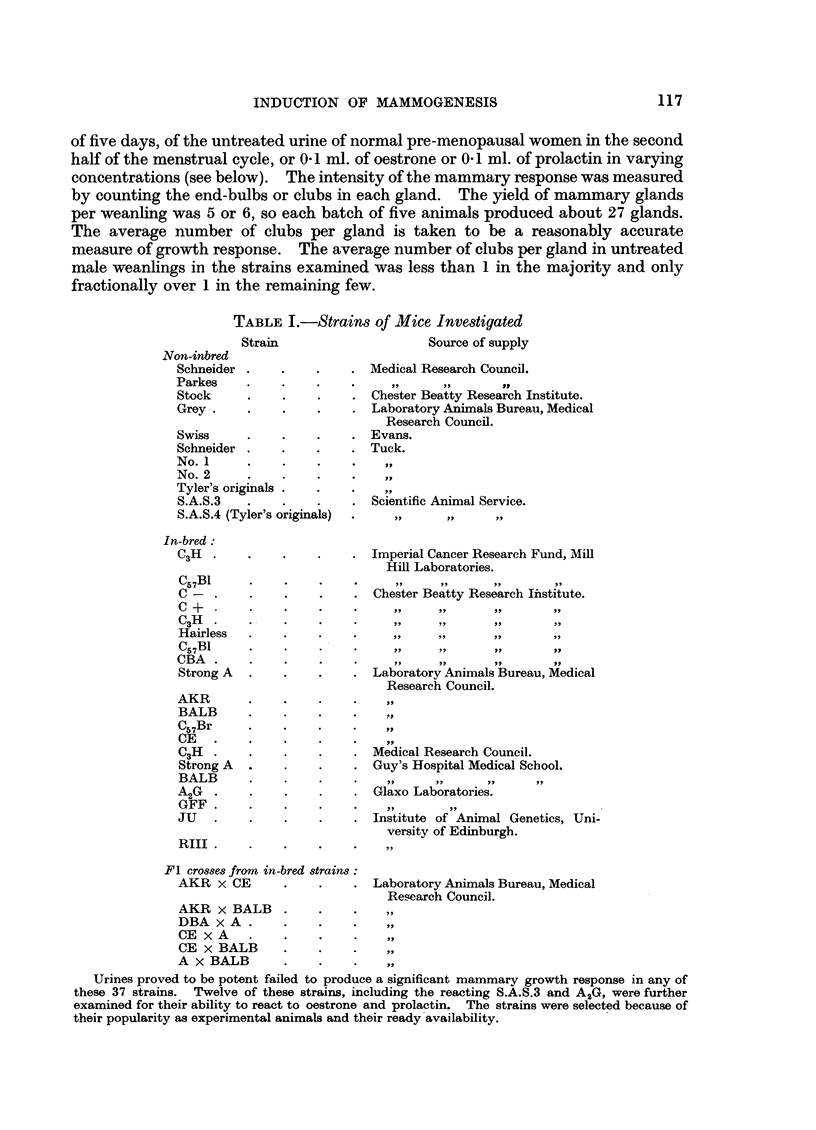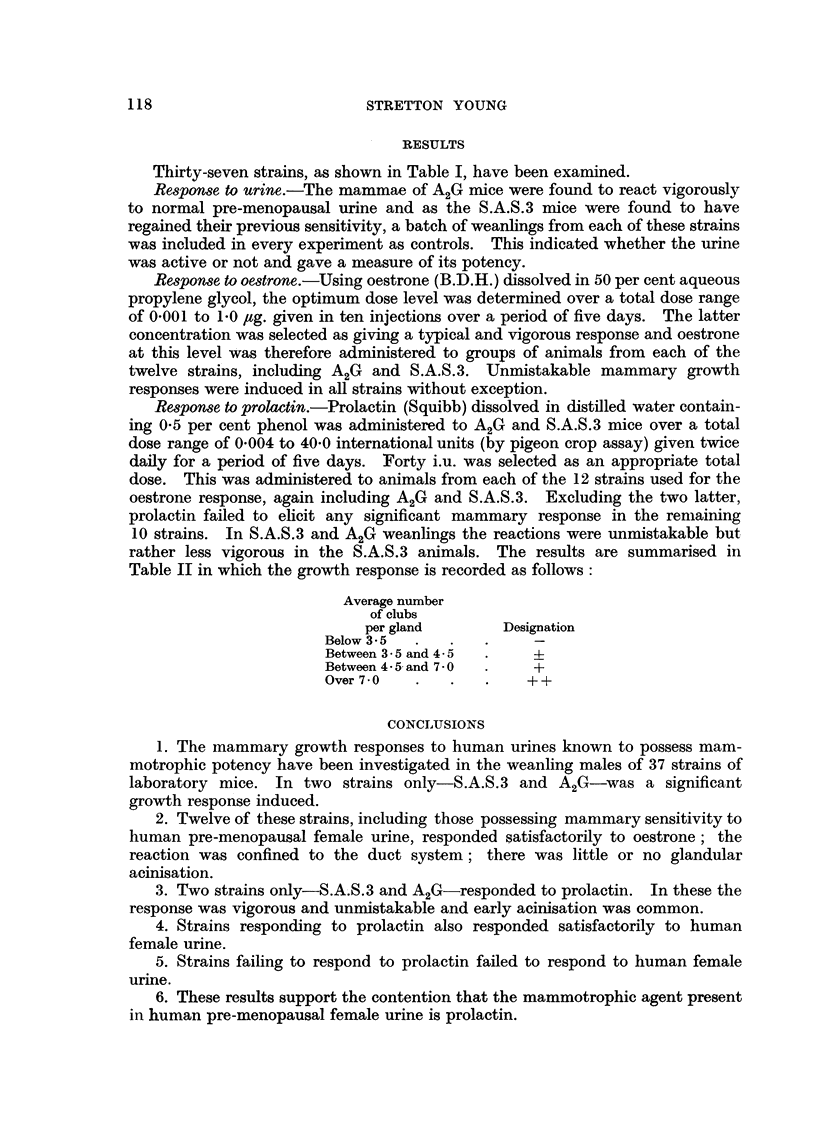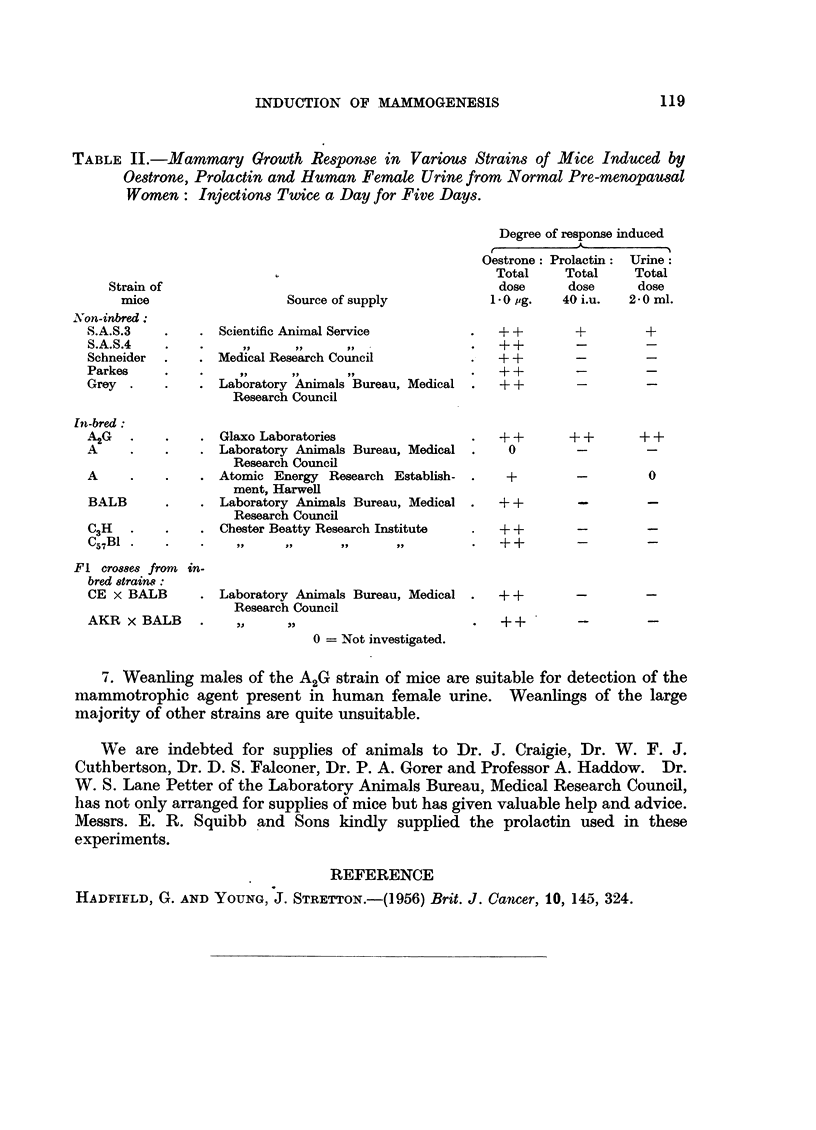# Induction of Mammogenesis by Prolactin and by Human Urine

**DOI:** 10.1038/bjc.1957.17

**Published:** 1957-03

**Authors:** Stretton Young


### INDUCTION OF MAMMOGENESIS BY PROLACTIN AND BY HUMAN URINE

STRETTON YOUNG

*From the Clinico-pathological Laboratories of the Imperial Cancer Research Fund, Lincoln's Inn Fields, London, W.C.2*

Received for publication February 1, 1957

HIGHLY significant growth responses in the rudimentary mammary glands of intact weanling male mice of the S.A.S.3 strain have been induced by pituitary prolactin when administered twice daily for five days by subcutaneous injection (Hadfield and Young, 1956). Compared with the responses produced by oestrone in the same strain at various dose levels over the same short period of injection, the mammae of prolactin-treated weanling males showed a significantly greater degree of glandular differentiation with the production of easily recognisable acini. Both hormones induced rapid development of the mammary ducts and ductules.

During the last two years, and until a few months ago, we have injected, twice daily for five days, a large number of specimens of human female urine into male weanlings of the S.A.S.3 strain, no other strain being used. Unmistakable mammary growth reactions have been obtained with the majority of urine specimens and these show the same degree of duct development and glandular differentiation which characterises the weanling mammae reacting to prolactin. Similar reactions to urine were obtained when specimens from normal pre-menopausal women in the post-ovulatory phase of menstruation were used, and significant responses were induced by the urine of 57 per cent of post-menopausal women.

Some months ago the mammary glands of the weanling males of the S.A.S.3 strain showed a precipitate fall in their ability to react both to prolactin and to human female urine (this has since returned). An extensive search was therefore made for another strain whose weanling males are sensitive to prolactin and urine. This has proved successful but the enquiry revealed the disconcerting fact that in the large majority of common strains of laboratory mice the mammary glands of the weanling males are quite unresponsive to prolactin and that this characteristic always appears to be associated with complete mammary insensitivity to human female urine. This observation explains the failure of several workers to confirm our published experiments which demonstrated the mammogenic property of human female urine when the " sensitive " mamma of S.A.S.3 weanling males was used as the " indicator ".

Other relevant facts have emerged from this enquiry. They throw some light on the nature of the mammotrophic hormone present in the urine of normal women and the present paper is a record of experiments dealing with these problems.

### METHODS

For each experiment five or more weanling males, aged 25 days on the day of the first injection, were used. They were given 0·2 ml. twice a day for a period of five days, of the untreated urine of normal pre-menopausal women in the second half of the menstrual cycle, or 0·1 ml. of oestrone or 0·1 ml. of prolactin in varying concentrations (see below). The intensity of the mammary response was measured by counting the end-bulbs or clubs in each gland. The yield of mammary glands per weanling was 5 or 6, so each batch of five animals produced about 27 glands. The average number of clubs per gland is taken to be a reasonably accurate measure of growth response. The average number of clubs per gland in untreated male weanlings in the strains examined was less than 1 in the majority and only fractionally over 1 in the remaining few.

TABLE I.—*Strains of Mice Investigated*

| Strain | Source of supply |
|---|---|
| *Non-inbred* | |
| Schneider | Medical Research Council. |
| Parkes | ,, ,, ,, |
| Stock | Chester Beatty Research Institute. |
| Grey | Laboratory Animals Bureau, Medical Research Council. |
| Swiss | Evans. |
| Schneider | Tuck. |
| No. 1 | ,, |
| No. 2 | ,, |
| Tyler's originals | ,, |
| S.A.S.3 | Scientific Animal Service. |
| S.A.S.4 (Tyler's originals) | ,, ,, ,, |
| *In-bred :* | |
| $C_3H$ | Imperial Cancer Research Fund, Mill Hill Laboratories. |
| $C_{57}Bl$ | ,, ,, ,, ,, |
| C − | Chester Beatty Research Institute. |
| C + | ,, ,, ,, ,, |
| $C_3H$ | ,, ,, ,, ,, |
| Hairless | ,, ,, ,, ,, |
| $C_{57}Bl$ | ,, ,, ,, ,, |
| CBA | ,, ,, ,, ,, |
| Strong A | Laboratory Animals Bureau, Medical Research Council. |
| AKR | ,, |
| BALB | ,, |
| $C_{57}Br$ | ,, |
| CE | ,, |
| $C_3H$ | Medical Research Council. |
| Strong A | Guy's Hospital Medical School. |
| BALB | ,, ,, ,, ,, |
| $A_2G$ | Glaxo Laboratories. |
| GFF | ,, ,, |
| JU | Institute of Animal Genetics, University of Edinburgh. |
| RIII | ,, |
| *F1 crosses from in-bred strains :* | |
| AKR × CE | Laboratory Animals Bureau, Medical Research Council. |
| AKR × BALB | ,, |
| DBA × A | ,, |
| CE × A | ,, |
| CE × BALB | ,, |
| A × BALB | ,, |

Urines proved to be potent failed to produce a significant mammary growth response in any of these 37 strains. Twelve of these strains, including the reacting S.A.S.3 and $A_2G$, were further examined for their ability to react to oestrone and prolactin. The strains were selected because of their popularity as experimental animals and their ready availability.

### RESULTS

Thirty-seven strains, as shown in Table I, have been examined.

*Response to urine.*—The mammae of $A_2G$ mice were found to react vigorously to normal pre-menopausal urine and as the S.A.S.3 mice were found to have regained their previous sensitivity, a batch of weanlings from each of these strains was included in every experiment as controls. This indicated whether the urine was active or not and gave a measure of its potency.

*Response to oestrone.*—Using oestrone (B.D.H.) dissolved in 50 per cent aqueous propylene glycol, the optimum dose level was determined over a total dose range of 0·001 to 1·0 $\mu$g. given in ten injections over a period of five days. The latter concentration was selected as giving a typical and vigorous response and oestrone at this level was therefore administered to groups of animals from each of the twelve strains, including $A_2G$ and S.A.S.3. Unmistakable mammary growth responses were induced in all strains without exception.

*Response to prolactin.*—Prolactin (Squibb) dissolved in distilled water containing 0·5 per cent phenol was administered to $A_2G$ and S.A.S.3 mice over a total dose range of 0·004 to 40·0 international units (by pigeon crop assay) given twice daily for a period of five days. Forty i.u. was selected as an appropriate total dose. This was administered to animals from each of the 12 strains used for the oestrone response, again including $A_2G$ and S.A.S.3. Excluding the two latter, prolactin failed to elicit any significant mammary response in the remaining 10 strains. In S.A.S.3 and $A_2G$ weanlings the reactions were unmistakable but rather less vigorous in the S.A.S.3 animals. The results are summarised in Table II in which the growth response is recorded as follows :

| Average number of clubs per gland | Designation |
|---|---|
| Below 3·5 | — |
| Between 3·5 and 4·5 | ± |
| Between 4·5 and 7·0 | + |
| Over 7·0 | ++ |

### CONCLUSIONS

1. The mammary growth responses to human urines known to possess mammotrophic potency have been investigated in the weanling males of 37 strains of laboratory mice. In two strains only—S.A.S.3 and $A_2G$—was a significant growth response induced.

2. Twelve of these strains, including those possessing mammary sensitivity to human pre-menopausal female urine, responded satisfactorily to oestrone ; the reaction was confined to the duct system ; there was little or no glandular acinisation.

3. Two strains only—S.A.S.3 and $A_2G$—responded to prolactin. In these the response was vigorous and unmistakable and early acinisation was common.

4. Strains responding to prolactin also responded satisfactorily to human female urine.

5. Strains failing to respond to prolactin failed to respond to human female urine.

6. These results support the contention that the mammotrophic agent present in human pre-menopausal female urine is prolactin.

TABLE II.—*Mammary Growth Response in Various Strains of Mice Induced by Oestrone, Prolactin and Human Female Urine from Normal Pre-menopausal Women : Injections Twice a Day for Five Days.*

| Strain of mice | Source of supply | Degree of response induced: Oestrone : Total dose 1·0 µg. | Prolactin : Total dose 40 i.u. | Urine : Total dose 2·0 ml. |
|---|---|---|---|---|
| *Non-inbred :* | | | | |
| S.A.S.3 | Scientific Animal Service | ++ | + | + |
| S.A.S.4 | " " " | ++ | – | – |
| Schneider | Medical Research Council | ++ | – | – |
| Parkes | " " " | ++ | – | – |
| Grey | Laboratory Animals Bureau, Medical Research Council | ++ | – | – |
| *In-bred :* | | | | |
| $A_2G$ | Glaxo Laboratories | ++ | ++ | ++ |
| A | Laboratory Animals Bureau, Medical Research Council | 0 | – | – |
| A | Atomic Energy Research Establishment, Harwell | + | – | 0 |
| BALB | Laboratory Animals Bureau, Medical Research Council | ++ | – | – |
| $C_3H$ | Chester Beatty Research Institute | ++ | – | – |
| $C_{57}Bl$ | " " " " | ++ | – | – |
| *F1 crosses from in-bred strains :* | | | | |
| CE × BALB | Laboratory Animals Bureau, Medical Research Council | ++ | – | – |
| AKR × BALB | " " | ++ | – | – |

0 = Not investigated.

7. Weanling males of the $A_2G$ strain of mice are suitable for detection of the mammotrophic agent present in human female urine. Weanlings of the large majority of other strains are quite unsuitable.

We are indebted for supplies of animals to Dr. J. Craigie, Dr. W. F. J. Cuthbertson, Dr. D. S. Falconer, Dr. P. A. Gorer and Professor A. Haddow. Dr. W. S. Lane Petter of the Laboratory Animals Bureau, Medical Research Council, has not only arranged for supplies of mice but has given valuable help and advice. Messrs. E. R. Squibb and Sons kindly supplied the prolactin used in these experiments.

### REFERENCE

HADFIELD, G. AND YOUNG, J. STRETTON.—(1956) *Brit. J. Cancer*, **10**, 145, 324.